# Staphylococcal Superantigens: Pyrogenic Toxins Induce Toxic Shock

**DOI:** 10.3390/toxins11030178

**Published:** 2019-03-23

**Authors:** Teresa Krakauer

**Affiliations:** Department of Immunology, Molecular Translational Sciences Division, United States Army Medical Research Institute of Infectious Diseases, Fort Detrick, Frederick, MD 21702 5011, USA; teresa.krakauer.civ@mail.mil

**Keywords:** staphylococcal superantigens, SEB, toxic shock, inflammation, damage response, therapeutics

## Abstract

Staphylococcal enterotoxin B (SEB) and related superantigenic toxins produced by *Staphylococcus aureus* are potent activators of the immune system. These protein toxins bind to major histocompatibility complex (MHC) class II molecules and specific Vβ regions of T-cell receptors (TCRs), resulting in the activation of both monocytes/macrophages and T lymphocytes. The bridging of TCRs with MHC class II molecules by superantigens triggers an early “cytokine storm” and massive polyclonal T-cell proliferation. Proinflammatory cytokines, tumor necrosis factor α, interleukin 1 (IL-1), IL-2, interferon γ (IFNγ), and macrophage chemoattractant protein 1 elicit fever, inflammation, multiple organ injury, hypotension, and lethal shock. Upon MHC/TCR ligation, superantigens induce signaling pathways, including mitogen-activated protein kinase cascades and cytokine receptor signaling, which results in NFκB activation and the phosphoinositide 3-kinase/mammalian target of rapamycin pathways. In addition, gene profiling studies have revealed the essential roles of innate antimicrobial defense genes in the pathogenesis of SEB. The genes expressed in a murine model of SEB-induced shock include intracellular DNA/RNA sensors, apoptosis/DNA damage-related molecules, endoplasmic reticulum/mitochondrial stress responses, immunoproteasome components, and IFN-stimulated genes. This review focuses on the signaling pathways induced by superantigens that lead to the activation of inflammation and damage response genes. The induction of these damage response genes provides evidence that SEB induces danger signals in host cells, resulting in multiorgan injury and toxic shock. Therapeutics targeting both host inflammatory and cell death pathways can potentially mitigate the toxic effects of staphylococcal superantigens.

## 1. Staphylococcal Exotoxins as Superantigens

*Staphylococcus aureus* is a ubiquitous Gram-positive coccus that produces several exotoxins with potent immunostimulating activities, which contribute to its ability to cause disease in humans, including food poisoning, skin infections, pharyngitis, acute lung injury, and toxic shock [1,2,3,4,5,6,7,8]. The bacterium readily colonizes humans via many virulence factors that promote bacterial survival and subsequent dissemination. Virulence factors such as leukocidins and α-hemolysin are cytotoxic to host cells [9]. Immunoevasive proteins include the C3 convertase blocker staphylococcal complement inhibitor (SCIN), which inhibits complement function [10] and chemotaxis inhibitory protein of *S. aureus* (CHIPS), which blocks formylated peptide recognition by the neutrophil receptor [11]. A large family of structurally related toxins, staphylococcal enterotoxins (SEs), and toxic shock syndrome toxin 1 (TSST-1), are the most potent due to their ability to polyclonally activate T-cells at picomolar concentrations [12,13,14,15,16,17,18]. Whereas TSST-1 and SEs activate macrophages and T-cells, SE-like (SEl) and staphylococcal superantigen-like (SSL) proteins exhibit various immunomodulatory activities [17,18,19]. SEl proteins are non-enterotoxic superantigens from *S. aureus*, but SSL proteins lack T-cell mitogenicity. For example, the SE-like protein SElX inhibits neutrophil phagocytosis, but is also capable of activating T-cells [18,19]. SSL proteins elicit activities against neutrophil and aid bacterial survival through evasion of the innate host defense.

The term “superantigen”, commonly used for SEs, TSST-1, and structurally related streptococcal pyrogenic exotoxins (SPEs) of *Streptococcus pyogenes*, was first coined by Kappler and Marrack in the late 1980s [12,13] to define microbial proteins that activate a large population (5–30%) of specific T-cells at picogram levels. Superantigens are in striking contrast to “conventional” antigens that normally stimulate <0.01% of T-cells at much higher concentrations [12,13,14,15]. Interactions between superantigens and host cells differ from conventional antigens in that superantigens (1) bind directly outside the peptide-binding groove of major histocompatibility complex (MHC) class II, (2) exert biological effects as an intact molecule without internalization and “processing”, and (3) are not MHC class II restricted. However, allelic differences exist in MHC class II binding affinities to superantigens and presentation to T-cells. For example, human HLA-DR binds staphylococcal enterotoxin B (SEB) and TSST-1 better than HLA-DQ or HLA-DP [20,21,22]. Human HLA-DR also binds bacterial superantigens with higher affinity than murine -IA and -IE [23]. Additionally, recognition of a superantigen and MHC class II complex by a T-cell receptor (TCR) depends upon the variable region within a TCR β chain (Vβ) [4,13]. Each superantigen binds to a distinct repertoire of TCR Vβ, thus revealing the unique Vβ specificities of an individual toxin [4,24].

By interacting with both MHC class II molecules on antigen-presenting cells (APCs) and specific elements within the variable region of the Vβ chains of a TCR, these microbial toxins perturb the immune system and induce high levels of proinflammatory cytokines and chemokines [12,13,14,15,16,17,25,26,27,28,29,30,31]. Other tissue-damaging molecules, such as matrix metalloproteinases (MMPs) and tissue factor, are also produced by superantigen-activated host cells, affecting both inflammatory and coagulation pathways [32]. Activated neutrophils produce reactive oxygen species (ROS), which leads to increased vascular permeability and lung injury [33]. Tumor necrosis factor α (TNFα) and interleukin 1 (IL-1) are induced early and are direct mediators of fever, hypotension, and shock [25,26,27,28,29,30,31]. In addition, IFNγ produced by activated T-cells acts synergistically with TNFα and IL-1 to enhance host defense by establishing an inflammatory environment for T-cell activation and differentiation [34]. Recently, another potent pathogenic cytokine, IL-17A, produced by CD4^+^ effector memory T-cells, was found to be rapidly induced in human PBMC exposed to SEA or SEB [35,36]. In vivo, the blockade of IL17A receptor signaling also reduced mortality, hepatotoxicity, and mucosal damage in a transgenic mouse model of toxic shock syndrome [36]. Since IL-17 has known proinflammatory effects and induces tissue damage in various autoimmune diseases [37], the early induction of IL-17 likely contributes to organ damage similar to IL-1.

Staphylococcal enterotoxin B has historically been the most intensively studied superantigen, and is listed as a category B select agent by the U.S. Centers for Disease Control and Prevention, as it can be used as an air-borne, food-borne, and water-borne toxin [38]. Depending on the dose and route of exposure, SEB and other SEs can cause food poisoning, acute and fatal respiratory distress, and toxic shock [2,3,4,5,6,38]. Staphylococcal superantigens also enhance the proinflammatory response and lethality by synergizing with other bacterial products, such as lipopolysaccharide (LPS), lipoproteins, and viruses [39,40,41,42,43]. Additionally, superantigens upregulate toll-like receptor 2 (TLR2) and TLR4, receptors that bind pathogen-associated molecular patterns (PAMPs), further amplifying the immune response to other microbial products [44,45]. Because it is common to encounter pathogens and their toxins concomitantly in real life, superantigens can have profound toxic effects at extremely low concentrations.

## 2. Receptor Binding and Cell Activation

Staphylococcal superantigens are stable, single-chain globular proteins of 22 to 30 kD that are highly resistant to proteases and heat denaturation [46,47]. Despite differences in amino acid sequence homology among SEs and TSST-1, they have similarities in their secondary and tertiary structures [15,17,48]. Cross-reactivities of polyclonal and monoclonal antibodies to various SEs and TSST-1 indicate common epitopes among these bacterial superantigens [49,50]. Crystallographic studies of staphylococcal superantigens have revealed two conserved, tightly packed domains with a β-barrel domain at the N-terminal and a C-terminal β-grasp motif [48]. The relatively conserved TCR binding site is located in the shallow groove separating these two domains [51]. The binding motifs of bacterial superantigens with MHC II and TCRVβ were defined by elegant structural and molecular studies in the 1990s and early 2000s [15,48,51]. Superantigens bind to common, conserved elements outside the peptide-binding groove on MHC II molecules with relatively high affinity [17,20,48]. There are at least two distinct binding sites on MHC II molecules for superantigens [52,53,54]. A common, overlapping, low-affinity generic binding site involving the invariant α-chain of MHC II is used by most superantigens [39,44]. A second high-affinity, zinc-dependent binding site on the polymorphic β-chain is used by some superantigens, such as SEA and SED [42,43,44,45,46,47,48,49,50,51,52,53,54]. SEA binds to both MHC II binding sites, and the cross-linking of MHC class II molecules on APCs by SEA activates monocytes, inducing potent proinflammatory mediators [55]. The mitogenic potency of superantigens is enhanced by a cooperative binding process such that the superantigen/MHC II complex binds the TCR with a higher affinity than a superantigen alone does [56]. The bridging of superantigen to MHC II and the TCR allows cooperative interactions between receptors, including costimulatory receptors, which further activates the host immune system [57]. 

## 3. Signal Transduction Pathways Induced by Superantigens

**Figure 1:**
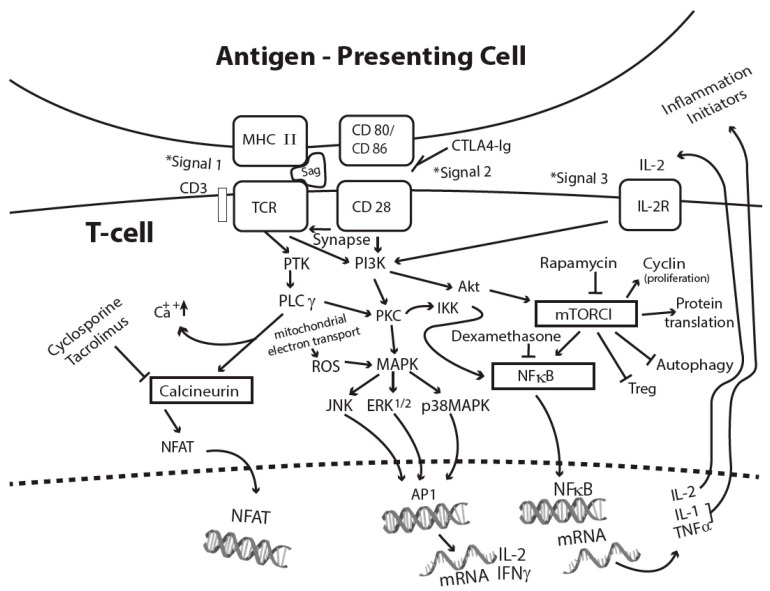
Superantigen-activated T-cell signaling pathways and sites of action of FDA-approved immunosuppressants. Abbreviations used: AP1, activating protein 1; CTLA4-Ig, cytotoxic T lymphocyte antigen-4 immunoglobulin; ERK1/2, extracellular signal-regulated kinase 1 and 2; IKK, IκB kinase; IL-2R, interleukin 2 receptor; JNK, jun-N-terminal kinase; MHC II, major histocompatibility complex class II; MAP, mitogen-activated protein kinase; mTORC1, mammalian target of rapamycin complex 1; NFAT, nuclear factor of activated T-cells; NFκB, nuclear factor kappa B; PI3K, phosphoinositide 3 kinase; PKC, protein kinase C; PLCγ, phospholipase C γ; PTK, protein tyrosine kinases; ROS, reactive oxygen species; Treg, regulatory T-cells; TCR, T-cell receptor.

The binding of superantigen/MHC II to a TCR transmits the classical first signal for T-cell activation [58,59]. Subsequent binding of costimulatory molecules CD80 and CD86 on APCs with CD28 on T-cells delivers the second signal, which optimizes T-cell activation through the formation of stable cell conjugates and supramolecular clusters [60,61]. Other cell-surface adhesion molecules and receptors such as CD2, intercellular adhesion molecule 1 (ICAM1), and endothelial leukocyte adhesion molecule (ELAM) facilitate the optimal activation of endothelial cells and T-cells by SEB [62]. CD11a/ICAM1 and CD28/CD80 costimulation also promotes SEA-mediated T-cell activation [60]. Costimulatory signaling enhances mRNA stability of IL-2, IFNγ, granulocyte-macrophage colony-stimulating factor (GMCSF), and the expression of antiapoptotic protein Bclxl to promote T-cell survival [63,64]. TCRs and costimulatory receptors activate protein tyrosine kinases (PTKs), LCK and ZAP-70, resulting in phospholipase C gamma (PLCγ) activation, the release of intracellular second messengers, and an increase in intracellular calcium [58,59,65]. Intracellular calcium concentration increase activates calcineurin phosphatase, which dephosphorylates the nuclear factor of activated T-cells (NFAT), allowing it to translocate into the nucleus, where it activates the expression of IL-2 and other T-cell cytokines [59,60,61]. Additionally, PTKs also activate protein kinase C (PKC) and the protooncogene Ras, both of which are also triggered by cell stress and growth factors [59]. The activation of PTK, PLCγ, and PKC initiate other signaling cascades, including mitogen-activated protein kinase (MAPK), extracellular signal-regulated kinase (ERK), and cJun N-terminal kinase (JNK). Cell activation culminates in the activation of transcriptional factors NFAT, AP1 (activating protein 1), and NFκB [58,59,66,67]. Nuclear NFκB binds to the promoter region of many proinflammatory mediators, including IL-1 and TNFα, resulting in proinflammatory cytokine expression [67]. The third signal to fully activate T-cells consists of inflammatory cytokines, T-cell growth, and differentiation factors, some of which can be induced by signal 1 and signal 2 [68]. Figure 1 illustrates the three signals provided by an antigen-presenting cell to sustain T-cell activation by superantigens.

TCR and costimulatory receptor stimulation also activate the lipid kinase phosphoinositide 3 kinase (PI3K), generating several inositol phospholipids and ultimately activating Akt and PKCθ [66,69,70]. PKCθ phosphorylates CARMA1, resulting in the recruitment of Bcl10 and MALT1 to form a CBM complex [71]. The CBM complex activates the inhibitor of κB (IκB) kinase complex (IKK) through a number of ubiquitin ligases. IKK phosphorylates IκB, releasing NFκB for nuclear translocation and gene activation [67,71]. NFκB regulates the transcription of genes for many cytokines, adhesion molecules, acute phase proteins, and antiapoptotic proteins Bcl2 and Bclxl [71]. In addition, IL-2 receptor (IL-2R), IFNR, growth factor receptors, and G-protein-coupled receptor (GPCR) also transduce activation signals upon ligand binding via the PI3K pathway. PI3K activation generates several inositol phospholipids and activates the protein kinase Akt and the mammalian target of rapamycin complex 1 (mTORC1) downstream [69,72]. Activation of mTORC1 from T-cell activation and the other receptors mentioned above promotes G1 to an S-phase transition, as it controls cell proliferation and protein translation [72]. However, mTORC1 activation also results in the suppression of autophagy [72], which is a homeostatic and catabolic process for lysosomal degradation of damaged organelles, protein aggregates, and intracellular pathogens [73].

## 4. Cellular Response to Superantigens

Human peripheral blood mononuclear cells (PBMCs) are often used to study the cellular requirements for activation and subsequent cellular changes by superantigens, as these cells are responsive to picomolar concentrations of staphylococcal enterotoxins (SEs) and TSST-1 [25,74,75]. The cytokines IL-1, TNFα, IFNγ, IL-2, IL-6, and chemokines, specifically macrophage chemoattractant protein 1 (MCP-1), are induced early by superantigens in human PBMCs [25]. There is also a correlation between the induction of these cytokines and lethal superantigen-induced shock in murine models [27,28,31,33,40,76]. IL-1 and TNFα also activate other cells, including fibroblasts, epithelial cells, and endothelial cells, to prolong inflammation by inducing cell adhesion molecules and additional mediators from these cells to increase vascular permeability [34]. Tissue factor and MMPs induced by IL-1 and TNFα contribute to damage to the immune and cardiovascular system, resulting in multiorgan dysfunction and lethal shock. Superantigen-activated T-cells induce the prototypic T helper cell type 1 (TH1) cytokine IFNγ, which augments immunological responses by increasing MHC class II and adhesion molecule ICAM on APCs, epithelial cells, and endothelial cells [28,62]. IFNγ also upregulates TNFα and IL-1 receptors, thus synergizing with TNFα and IL-1 to promote tissue injury [34]. IFNγ and TNFα, individually and together, can also initiate a programmed necrotic cell death called necroptosis [77]. The receptors and signaling pathways for these mediators are diverse, accounting for the different immunomodulating activities of cytokines. The intracellular and molecular components of cytokine receptor signaling have been studied extensively, as they serve as targets of therapeutic interventions of various inflammatory diseases [78,79,80,81].

## 5. IL1β and Inflammasome Activation

IL-1β binds IL-1 receptor 1 (IL-1R1) and recruits an accessory protein to activate NFκB via the following signaling adaptors: Myeloid differentiation factor 88 (MyD88), IL-1R-associated protein kinase (IRAK), and TNF receptor-associated factor 6 (TRAF6) [81,82]. The IL-1R1 signaling pathway is highly conserved, and its signaling components are also triggered by the binding of PAMPs to pattern recognition receptors (PRRs) [83,84]. PAMPs such as lipoprotein, LPS, flagellin, dsRNA, and viral RNA bind to specific cell surfaces and endosomal toll-like receptors (TLRs) to activate the innate host response. A central component of IL-1R1/TLR signaling is the activation of IKK, resulting in nuclear translocation and activation of NFκB, which induces inflammation and cell survival. The IL-1R/MyD88/TRAF6 pathway also activates the stress kinases JNK and MAPK to promote cell stress and inflammation [34,67,84]. 

The production of IL-1β is a highly regulated process involving proteolytic cleavage of pro-IL-1β to mature, active IL-1β by active caspase 1. The autoproteolysis of pro-caspase 1 to active caspase 1 requires the activation and assembly of a multiprotein complex, which is known as an inflammasome [85,86]. The NLR family pyrin domain-containing protein 3 (NLRP3) inflammasome is the best-known inflammasome for IL-1β activation and release. NLRP3 can be activated by a diverse group of stimuli, including bacteria, viruses, fungi, pore-forming toxins, ATP, uric acid crystals, peptide aggregates, ROS, mitochondrial DNA, lysosomal ruptures, and potassium efflux [86,87]. Since these stimuli do not have a common structure, induced cellular perturbation is likely the critical factor that activates NLRP3. Concomitant with the activation of NLRP3 is the activation of pyroptosis, an inflammatory form of cell death that releases cellular contents and promotes inflammation and tissue injury [85].

## 6. TNFα Activates Inflammation and Cell Death

TNFα activates NFκB by binding to TNF receptor 1 (TNFR1) or TNFR2. The cytotoxic functions of TNFα are mostly mediated by its binding to TNFR1 via cytoplasmic death domains, which are absent in TNFR2 [34,80]. Thus, TNFR1 signaling produces inflammatory and pro-survival responses via NFκB and MAPK cascades and cell death (apoptosis and/or necroptosis) via caspase 8-dependent and independent pathways [80]. The induction of these pathways is interconnected through signaling complexes that are tightly controlled by mechanisms that are not fully understood [88,89]. Within seconds of TNFα binding to TNFR1, a membrane-bound signaling complex is formed with the recruitment of TNFR-associated death domain (TRADD), receptor-interacting protein kinase 1 (RIPK1), cellular inhibitor of apoptosis (cIAPs), and other components to trigger IKK and NFκB activation, resulting in inflammation and cell survival. Subsequently, other cytosolic signaling complexes can be formed to induce cell death, depending on the dissociation of TRADD and RIPK1. Apoptosis is induced by the formation of a complex consisting of Fas-associated death domain (FADD), RIPK1, and caspase 8. Inhibition of caspase 8 and the deubiquitylation of RIPK1 allows RIPK1 to interact with another serine/threonine kinase, RIPK3, through their RIP homotypic interaction motif (RHIM) domains for the assembly of the necrosome (RIPK1/RIPK3), resulting in RIPK3 phosphorylation and activation. RIPK3 then phosphorylates and activates the pseudokinase mixed lineage kinase domain-like (MLKL). Activated MLKL induces pore formation in the plasma membrane and cell leakage of damage-associated molecular patterns (DAMPs) [89]. DAMPs can be sensed by cytosolic PRRs to activate inflammasomes to release IL-1β, IL18, and pyroptosis [90]. The detection of DAMPs and PAMPs by the same cytosolic PRRs results in cell death, accounting for the similar pathogenic effects of superantigens and infectious agents [89,90]. The RIPK1/RIPK3/MLKL pathway of necroptosis contributes to tissue damage, cell death, and inflammation [91].

Apoptosis is a programmed process of packaging cell contents for elimination and is dependent on sequential proteolytic activation of caspases [92]. Apoptosis has homeostatic functions during development, infection, and the recovery phase of infection [93]. The extrinsic or intrinsic pathways of apoptosis are mediated by ligands binding to the TNFR superfamily or by mitochondrial damage, respectively [92]. Extrinsic apoptosis is dependent on caspase 8 activation, whereas intrinsic apoptosis is dependent on the activation of caspase 9. Both pathways subsequently activate caspase 3 and caspase 7 downstream. The TNFR superfamily activates the extrinsic pathway of apoptosis with the formation of the FADD/RIPK1/caspase 8 complex. Activation of caspase 8 and RIPK3 controls the balance of cell demise either by apoptosis or by necroptosis [88]. The intrinsic pathway of apoptosis is activated by mitochondrial ROS and damage [92]. Apoptotic cells display many recognition receptors for their removal by phagocytes [93]. Apoptosis is normally a noninflammatory form of cell death [93,94]. However, uncleared apoptotic cells eventually become necrotic and release cellular DAMPs to promote tissue injury. A number of recent studies have revealed that the apoptotic caspase 8 has many other activities, including the suppression of necroptosis by cleavage of RIPK1, RIPK3, and caspase 1; proteolytic processing of IL-1β to its mature active form; priming NLRP3; and the regulation of cytokine transcriptional responses [95,96]. The crossover inflammatory activities of caspase 8 indicates that caspase 8 can act as an inflammasome, depending on its catalytic action on caspase 1, IL-1β, RIPK1, and RIPK3, as well as the absence or depletion of caspase inhibitors.

TNFR1 signaling activates MAPK, NFκB, apoptosis, and necroptosis, accounting for the pleiotropic effects of TNFα, including cell activation, cell death, coagulation, inflammation, and antimicrobial defense [34,80]. The activation of NFκB to promote inflammation and cell survival is induced with faster kinetics by TNFR1 signaling. TNFR1-induced intracellular complexes leading to cell death occur later and are tightly regulated by activities of caspase 8, RIPK1, and the presence of cellular inhibitors of apoptosis [88].

The TNFR superfamily member Fas (CD95) and TRAIL death receptors also share similar signaling complexes with TNFR1 to induce apoptosis and necroptosis after the binding of their respective ligands [80,88]. SEA upregulates the expression of Fas and induces apoptosis via caspase 8 activation [97]. Necroptosis is known to occur in superantigen-induced toxic shock, as pore-inducing MLKL was expressed in blood leukocytes and various organs including the lungs, kidneys, and heart in a murine model of SEB-induced shock [98].

## 7. IFN Signaling Contributes to Cell Death

IFNγ (type II IFN) is produced by NK cells, CD8 T-cells, and the TH1 subset of CD4 T-cells. IFNγ binds to IFNGR1 and signals via Janus kinase 1 (JAK1), JAK2, and signal transducer and activator of transcription 1 (STAT1) [99,100]. Both type I (IFNα and IFNβ) and type II IFNs signal via PI3K after binding to their respective receptors (IFNAR1 and IFNGR1). Although the main function of type I IFN is antiviral, IFNα and IFNβ have many overlapping activities with IFNγ, as they induce many common interferon-stimulated genes (ISGs) [100]. ISGs have antiviral, antiangiogenic, and ubiquitylating activities. The immunomodulatory and antimicrobial effects of IFNs are mediated by immunity-related GTPase (IRGs) and guanylate binding proteins (GBPs) [100,101]. IFNγ also induces immunoproteasomes and the expression of MHC class II molecules to enhance antigen processing and improve the adaptive immune response [100]. Immunoproteasome components degrade apoptotic inhibitors and contribute to vascular cell apoptosis and cardiovascular inflammation [102]. IRGs, GBPs, immunoproteasomes, and other ISGs were highly expressed in blood leukocytes and multiple organs in a mouse model of toxic shock [98].

IFNγ activates the PI3K/AKt/mTORC1 pathway and PKC, resulting in protein translation and MAPK activation. IFNs also induce apoptosis and necroptosis in macrophages [103,104]. Both types of IFNs induce and activate death receptors, such as Fas, which then activates FADD, subsequently activating caspase 8. Activated caspase 8 can cleave proapoptotic molecule Bid to a truncated form, allowing for its interaction with two mitochondrial proapoptotic molecules, Bak and Bax [105]. The oligomerization of Bak/Bax results in mitochondrial outer membrane permeabilization and the release of cytochrome c to the cytosol. Cytochrome c binds cytosolic protein apoptotic protease-activating factor 1 (APAF1), forming an apoptosome with the recruitment of caspase 9 [92]. Activation of both initiator caspases, caspase 8 or caspase 9, leads to the induction of effector caspases to execute apoptotic cell death programs. Damage to mitochondria also releases mitochondrial DNA (mtDNA), which has similar motifs to bacterial DNA and activates cytosolic DNA sensors [106,107,108]. SEA-induced hepatotoxicity is mediated by the Fas ligand (FasL), and the hepatocellular damage is independent of leukocyte recruitment [97]. TNFα and IFNγ act synergistically on epithelial cells to increase ion transport and disrupt epithelial barrier function [109,110]. IFNγ also synergizes with IL-1 and TNFα to promote leukocyte recruitment, inflammation, and coagulation [34].

The necroptotic activity of IFNγ is dependent on STAT1 and the eukaryotic translation initiation factor 2 alpha kinase 2 (EIF2AK2, also known as PKR) [104]. Similarly to TNFR1-induced necroptosis, the activation of MLKL by IFNγ is modulated by caspase 8, cellular inhibitors of apoptosis, and the RIPK3 activation status [88]. Two other RHIM-containing adaptors, Z-DNA binding protein 1 (ZBP1, also known as DAI) and Toll/IL-1 receptor domain-containing adaptor-inducing IFNβ (TRIF), also activate RIPK3 individually through homotypic RHIM interactions in response to TLR3 or TLR4 activation to promote necroptosis [111,112]. ZBP1, a known DNA sensor, was induced in multiple organs and blood leukocytes in a mouse model of SEB-induced shock [98]. Damaged mtDNA released as a result of oxidative stress binds ZBP1 and triggers inflammation in pulmonary epithelial cells [113].

## 8. Contributions by Other Cytokines and Chemokines

IL-2 activates T-cells by binding to high-affinity IL-2Rs [114]. It signals through JAK1 and JAK3, activating PI3K/Akt/mTORC1 and Ras to promote cell growth, differentiation, and proliferation [114]. Ras activates the MAPK and ERK cascades, leading to the activation of AP1 and NFAT. IL-2 from SEB-activated T-cells has potent vascular effects and induces vasodilation, vascular leak, and edema [29,115]. TNFα synergizes with IL-2 to promote vascular leak, as seen in acute lung injuries induced by superantigens or pathogens [33,115,116,117].

IL-6, from both macrophages and activated T-cells, has some overlapping activities with IL-1 and TNFα. IL-6R activates JAK3 and Ras upon ligand binding [118]. Activated JAK3 phosphorylates STAT3, which then dimerizes and translocates to the nucleus, where it binds target genes essential for cell survival and G1 to an S-phase transition. IL-6R also signals through PI3K/Akt/mTORC1 to promote cell survival. Together and individually, IL-1, TNFα, and IL-6 act on the liver to release acute phase proteins, increase coagulation, and compromise liver clearance activities [34].

The chemokines IL-8, MCP-1, macrophage inflammatory protein (MIP)-1α, and MIP-1β are induced directly by SEA, SEB, or TSST-1 [28,29,115,116]. These chemokines activate leukocytes and act as chemoattractants to influence the migration of neutrophils, dendritic cells, and leukocytes [34,119,120]. Chemokines bind to seven-transmembrane GPCRs, induce early calcium flux, activate PLC, and signal via the PI3K/Akt/mTORC1 pathway. Cytokine- and chemokine-activated neutrophils, recruited to sites of tissue injury and inflammation, produce ROS and activate MMPs contributing to organ damage [119]. MMPs cause tissue degradation and change chemokine interactions with the extracellular matrix, creating a local chemokine gradient affecting cell recruitment [119]. Exudates from superantigen-injected air pouches are predominantly neutrophils, with some macrophages [28]. Both systemic and intranasal exposure to SEB cause acute lung injury, characterized by increased expression of adhesion molecule ICAM-1 and vascular cell adhesion molecule (VCAM), increased neutrophils and mononuclear cell infiltrates, endothelial cell injury, and increased vascular permeability [28,33,115]. A blockade of ICAM-1 by anti-ICAM-1 antibodies attenuated pulmonary barrier damage in a murine model of SEA-mediated acute lung injury [121].

## 9. Oxidative Stress Damages Mitochondria and Releases DAMPs

Superantigens induce massive proliferation in resting T-cells, which requires increased protein synthesis and metabolic activities. Oxidative stress, ROS, MAPK, increased protein synthesis, and fatty acid oxidation as a result of cell activation promote endoplasmic reticulum (ER) stress [122,123]. SEB induces the expression of ubiquitin ligases, proteasome peptidases, and immunoproteasomes in multiple organs [98]. These ER stress response genes are likely a result of calcium flux and PKC activation from TCR stimulation by superantigens. Prolonged ER stress activates the unfolded protein response and apoptosis via the induction of caspases [124,125,126]. ER stress also activates NLRP3 [126,127] and induces IL-1β through a caspase 8-dependent pathway [128]. Increased activity of the mitochondrial electron transport chain following superantigen-activated proliferation also promotes oxidative stress and the generation of ROS, leading to mitochondrial damage. 

Hyperactivation of mTORC1 in superantigen-activated cells disrupts normal host autophagy. Mitophagy is a specialized form of autophagy which normally removes damaged mitochondria from oxidative damage and other cell stress signals [129,130,131]. Superantigens enhance mitochondrial respiration and the production of ROS which damage mitochondria, activate caspase 9, and promote apoptosis [73,105]. Damaged mitochondria release cytochrome c, ATP, N-formyl peptides (NFPs), and mtDNA to the cytosol in addition to activating apoptotic caspases via the intrinsic cell death pathway [108,130,131,132]. NFPs activate and attract neutrophils to sites of inflammation and infection, contributing to tissue damage [130]. MtDNA induces MMP8 and MMP9 secretion by activating p38MAPK [131]. In addition, mtDNA binds endosomal TLR9, activating the transcriptional factors NFκB and interferon-regulatory factor 7 (IRF7). The leakage of mtDNA by damaged mitochondria as a result of oxidative stress exacerbates inflammation as mtDNA acts as a potent DAMP to activate TLR9 and ZBP1, critical sensors of “danger” in the detection of intracellular pathogens.

## 10. DAMPs and Inflammatory Cytokines Promote Cell Death and Inflammation

DAMPs such as ROS and mtDNA are upstream activators of inflammasomes and induce inflammatory cytokines and pyroptosis [73,86,131,132,133,134]. ER stress, viral entry, and replication can destabilize lysosomes and activate inflammasomes [86,126]. MtDNA binding to ZBP1 induces IFNs to promote necroptosis and inflammation [106,130]. Mitochondria1 DAMPs synergize with each other and with cytokines to promote inflammation [130]. Inflammasome activation and TNFR1 signaling contribute to pyroptosis and necroptosis, respectively. Inflammasome activation, in addition, activates caspase 1 to release inflammatory cytokines. TNFR1 signaling induces inflammation by activating the MAPK cascade and NFκB [123,133]. IFNγ triggers innate host defense responses, antiviral genes, apoptotic programs, and immunoproteasomes, and has many immunomodulatory functions. The cell death pathways triggered in vitro and in vivo by superantigens include genes associated with apoptosis such as FADD, death receptor ligand TRAIL (TNFSF10), caspases, and phospholipid scramblase 1 (PLSCR1) [98,135]. DNA damage repair enzymes (poly [ADP-ribose] polymerases) were induced in PBMCs and multiple organs of a mouse model of SEB-mediated shock, indicating DNA damage and repair [108]. Cellular injury is also apparent from the expression of MMPs, cathepsins, and other cell matrix breakdown products in superantigen-activated cells [98,135]. Necroptotic components MLKL, PKR, and ZBP1 were also upregulated in multiple organs and PBMCs in a mouse model of SEB-induced toxic shock [98].

Apoptosis plays a critical role in downregulating immune responses, but simultaneously has detrimental effects when apoptotic cell death is unrestrained, as uncleared apoptotic cells become necrotic [93,94]. Autophagy serves to maintain cellular homeostasis by removing protein aggregates and damaged organelles [136]. In particular, mitophagy reduces mtDNA and other mitochondrial DAMPs [73,129,136]. Autophagy-dependent degradation and removal of Bcl10, which is part of the CBM complex for TCR and costimulatory signaling, downregulates T-cell activation [137]. In superantigen-activated cells, autophagy likely protects cells by removing DAMPs and Bcl10 and downregulates inflammation.

## 11. Lessons Learned from Therapeutics That Prevent SEB-Induced Shock

Limited therapeutics for treating superantigen-induced toxic shock are currently available. Intravenous immunoglobulin has been effective as a treatment in humans after the onset of toxic shock syndrome [1,138]. Antibody-based therapy targeting direct neutralization of SEB or other superantigens is most suitable during the early stages of exposure, before cell activation and the induction of proinflammatory cytokines. Various humanized monoclonal antibodies that have been developed can neutralize SEs and TSST-1 by targeting specific epitopes on SEs and TSST-1 [139,140,141]. A mixture of antibodies will likely be effective in treating exposures to a greater variety of superantigens.

Potential targets to prevent the toxic effects of staphylococcal superantigens include (1) blocking the interaction of superantigens with MHC, TCRs, or other costimulatory molecules; (2) inhibiting the signal transduction pathways initiated by superantigens; and (3) inhibiting cytokine and chemokine production and their signaling pathways. This topic will be summarized briefly in the context of U.S. Food and Drug Administration (FDA)-approved drugs that have been tested in animal models of superantigen-induced shock [142]. Drugs inhibiting superantigen and receptor interactions have to be administered early upon toxin exposure, which is not always possible. The most effective mode of intervention is a blockade of signal transduction pathways and molecules as signaling occurs postexposure, and a downstream blockade will likely be effective against other superantigens. 

Both NFκB and mTORC1 are prime targets for intervention, as they are key hubs of signal transduction, mediating the major biological responses to superantigens from TCR, costimulator CD28, and cytokine signaling. NFκB initiates key mediators of inflammation and pro-survival signals, whereas mTORC1 promotes protein translation and inhibits autophagy. IL-1β, TNFα, and IFNγ have independent and synergistic effects to induce inflammation and various forms of cell death. Cell activation induces ER stress, oxidative stress, mitochondrial ROS, and damage, which also promotes cell death. An upregulation of damage response genes contributes to the irreversible multiorgan damage seen in animal models of superantigen-induced toxic shock and human toxic shock syndrome [33,98,115,121,143,144,145]. In this regard, superantigen-induced mTORC1 activation increases oxidative stress, contributing to inflammation, DAMPs, and cell death.

## 12. Mouse Models of Superantigen-Induced Shock

Mice are often used as a model to study the immunological mechanisms of superantigen-mediated shock [27,28,31,33,40,41,42,43]. They are ideal to work with regarding costs for in vivo screening of potential vaccines and therapeutics. However, mice are naturally less susceptible to SEs, TSST-1, and SPEs (versus humans) because of lower toxin affinity to murine MHC class II. To overcome this last caveat, potentiating agents such as D-galactosamine, actinomycin D, lipopolysaccharide (LPS), or viruses have been used by various laboratories to amplify the toxic effects of superantigens in mice so that practical, lower amounts of toxins can be used for in vivo studies of toxic shock [27,31,40,41,42,43]. All of these studies have revealed a correlation between elevated serum levels of various proinflammatory cytokines (IL-1, IL-2, TNFα, and/or IFNγ) with SEA-, SEB-, or TSST-1-induced shock. These superantigen-induced shock murine models using potentiating agents have major drawbacks for therapeutic studies, as the sensitizing agents themselves often induce the same mediators as SEs or TSST-1 by activating similar cells and signaling pathways. Both actinomycin D and D-galactosamine are hepatotoxic, and mouse models using these potentiating agents produce unrealistically high levels of TNFα and liver damage [146]. Drugs designed to inhibit TNFα have a higher therapeutic impact in models using these two potentiating agents. In the SEB-plus-LPS mouse model, the synergistic action of SEB and LPS promotes early TNFα release and prolongs the release of IFNγ, IL-2, IL-6, and MCP-1 [31]. The higher and prolonged levels of these mediators lead to acute mortality, with mice succumbing to toxic shock within 48 h when LPS is used together with SEB [31,40]. Importantly, the lethal endpoint of these murine models is different from human and nonhuman primates exposed to SEB [38]. Two newer, simplified murine models have been developed to study SEB-induced shock without potentiating agents. Transgenic mice expressing human MHC class II respond to lower doses of SEB without synergistic agents due to the higher-affinity binding of SEB to human MHC class II molecules [147,148,149]. Transgenic mice with human HLA-DR3 or -DQ8 lethally respond to SEs without potentiation, and the serum levels of mediators correlate with lethal shock [148,149]. Pathological lesions in the lungs of transgenic mice, temperature fluctuations, and delayed lethal endpoints are similar to those in nonhuman primates exposed to lethal doses of SEB [38,149]. Low-dose continuous administration of SEB to HLA-DQ8 transgenic mice induces a lupus-like disease with multiple organ injury [78]. An alternative murine model deploys a “double-hit” strategy, with two low doses of SEB using the LPS-resistant C3H/HeJ mice [115]. This "SEB-only" toxic shock model relies on the intranasal administration of SEB and the enhanced action of another dose of SEB later to induce pulmonary inflammation and lethal shock. Importantly, pathological lesions, cytokine response, multiple organ injury, and time to lethality in this “SEB-only” model have resembled findings in nonhuman primates and clinical staphylococcal toxic shock syndrome [1,98]. Other animal models used to study the in vivo biological effects of bacterial superantigens include NHPs, piglets, and rabbits [38,150,151,152,153,154,155]. SEs readily induce an emetic response in primates when ingested in low-microgram quantities [150]. Classic primate studies for SEs have been performed by various groups and are considered the “gold standard”, as NHP models mimic different aspects of human disease induced by these superantigens. However, the high cost and animal welfare concerns associated with NHPs limit their use for routine therapeutic efficacy testing. Piglets have been used to study TSST-1-induced effects and more recently to test SEB vaccines [152,153]. Cows have been used to study mastitis induced by SEC [156] and T-cell repertoires responsive to bovine infective isolates producing superantigens [157]. Toxic shock models with rabbits using subcutaneous or continuous infusion of superantigens also mimic human disease closely, but a lack of biological reagents hampers their use for immunological studies [154,158]. Other toxic shock rabbit models have been developed recently to study infective endocarditis, acute kidney injury, and sepsis using *S. aureus*-producing various superantigens [18,144]. These rabbit models are invaluable in providing in vivo information for the development of sepsis and septic complications of bacterial infections.

## 13. FDA-Approved Drug Blockade of Superantigen-Induced Shock

Recently, a blockade of T-cell costimulatory signals by abatacept, a FDA-approved cytotoxic T-lymphocyte antigen-4 immunoglobulin (CTLA4-Ig), prevented SEB-induced lung damage in mice and reduced cytokine levels both in vitro and in vivo [159]. This was in agreement with a previous study using CTLA4-Ig to prevent toxic shock in a D-galactosamine-sensitized mouse model [160]. Inhibition of NFκB by dexamethasone, a potent corticosteroid, has been effective in preventing SEB-induced shock in various animal models [161,162]. However, the dexamethasone was effective only if administered soon after superantigen exposure and for a long duration [162]. The calcineurin inhibitors cyclosporine A (CsA) and tacrolimus (FK506) are known inhibitors of T-cell activation that have had contradictory effects against superantigens in vitro and in vivo [163,164,165]. CsA prevented SEB-induced shock in a D-galactosamine-sensitized mouse model of toxic shock [164]. Although CsA inhibited SEB-induced T-cell proliferation in vitro and attenuated pulmonary inflammation and serum cytokines, it had no effect on mortality in nonhuman primates [165]. Tacrolimus suppressed superantigen-induced T-cell activation in vitro, but was ineffective in reducing lethality in transgenic mice exposed to SEB [166]. Rapamycin, a mTORC1-specific inhibitor, blocked SEB-induced T-cell proliferation, as well as SEB-induced IL-2 and IFNγ production, in vitro and in vivo [167,168]. Importantly, inhibition of the SEB-induced PI3K/Akt/mTORC1 pathway by rapamycin prevented lethal toxic shock in a mouse model even if rapamycin was administered 17 h after SEB exposure. Rapamycin likely prevents organ damage by inducing autophagy via the inhibition of mTORC1. Rapamycin also suppresses inflammation by increasing the number of regulatory T-cells [169,170]. The removal of DAMPs by autophagy prevents cytosolic PRR activation and reduces inflammation and cell death [73,171]. The success of rapamycin in preventing SEB-induced toxic shock indicates that organ damage induced by a cytokine storm can be reduced by the upregulation of autophagy and regulatory T-cells. These homeostatic responses, initiated after excessive inflammation induced by superantigens, are critical in dampening inflammation and limiting organ injury.

## 14. Summary

Proinflammatory cytokines act synergistically on multiple cell types and mediate the toxic effects of staphylococcal superantigens. The concomitant activation of inflammasomes and mitochondrial DAMPs by superantigens initiates pyroptosis and necroptosis, contributing to multiorgan injury. The ability to inhibit the cytokine cascade early appears to be critical in mitigating the toxicity of staphylococcal superantigens. However, pharmacological intervention to block cell death and organ damage remains a challenge in preventing superantigen-induced toxic shock.
